# More than a trend: the rationale for using bioactive materials in clinical practice

**DOI:** 10.1590/1807-3107bor-2026.vol40suppl1060

**Published:** 2026-08-24

**Authors:** Igor Paulino Mendes SOARES, Maria Luiza Barucci Araújo PIRES, Juliana Rios de OLIVEIRA, Lídia de Oliveira FERNANDES, Carlos Alberto de Souza COSTA, Marco Cícero BOTTINO, Josimeri HEBLING

**Affiliations:** (a) Universidade Estadual Paulista – Unesp, School of Dentistry, Department of Dental Materials and Prosthodontics, Araraquara, SP, Brazil; (b) Universidade Estadual Paulista – Unesp, School of Dentistry, Department of Morphology, Orthodontics and Pediatric Dentistry, Araraquara, SP, Brazil; (c) Universidade Estadual Paulista – Unesp, School of Dentistry, Department of Restorative Dentistry, Araraquara, SP, Brazil; (d) Universidade Estadual Paulista – Unesp, School of Dentistry, Department of Physiology and Pathology, Araraquara, SP, Brazil; (e) University of Michigan, School of Dentistry, Department of Cariology, Restorative Sciences, and Endodontics, Ann Arbor, MI, USA

**Keywords:** Dentin, Dental pulp, Biocompatible Materials, Biomineralization

## Abstract

The concept of bioactivity in restorative dentistry has evolved beyond the traditional expectation of inert materials designed solely to replace lost tooth structure. Contemporary restorative systems are increasingly engineered to interact with the dentin-pulp complex by modulating mineral dynamics, matrix stability, and cellular responses. However, misconceptions remain regarding what restorative “bioactivity” can realistically achieve in clinical practice. This review critically discusses the biological basis and clinical relevance of bioactive materials in restorative dentistry, emphasizing dentin-pulp preservation and the distinction between validated bioactivity and laboratory observations. Bioactivity may arise through physicochemical mechanisms, such as ion release, pH modulation, and mineral precipitation, which may contribute to mineral recovery and dentin-pulp preservation, although many proposed effects remain insufficiently validated clinically. In parallel, biological mechanisms involve modulation of odontogenic signaling, pulp cell differentiation, and extracellular matrix dynamics. Strategies targeting collagen stability, including cross-linking, protease inhibition, and intrafibrillar mineralization, may also improve hybrid-layer durability and interfacial longevity. Beyond dentin-focused interventions, pulp-directed bioactivity includes clinically established vital pulp therapy materials and emerging scaffold-based regenerative systems that remain largely investigational. However, important translational questions remain regarding the clinical functionality of current materials. Clinically relevant restorative bioactivity should not be defined by ion release or surface mineral deposition alone, but by the ability of materials to preserve dentin-pulp homeostasis and long-term restorative stability. Moving beyond the “bioactive” label requires evidence-based strategies that sustain dentin-pulp homeostasis rather than merely replacing lost structure.

## Introduction

Traditionally, the development of restorative dental materials has focused on optimizing their adhesive and mechanical performance, relying on micromechanical and chemical bonding to achieve durable integration with dental substrates while reducing the need for macro-retentive preparations.^1^, ^2^ Resin composites, in particular, have been formulated to meet stringent demands related to aesthetics, mechanical strength, and long-term degradation resistance.^1^ These goals are achieved through the careful design of polymer matrices and reinforcing fillers that can sustain performance in the dynamic oral environment.^1^

Biocompatibility has also remained a key requirement, ensuring that these materials elicit an appropriate tissue response that optimizes the clinical performance of the therapy, while minimizing adverse reactions and supporting clinical longevity.^3^, ^4^ While biocompatibility refers primarily to the absence of harmful effects, bioactivity implies the ability of materials to actively induce beneficial biological interactions at the tissue interface.^2^, ^4^, ^5^, ^6^ Historically, restorative materials were viewed as functional but essentially passive systems, intended to reestablish form and function without actively modulating the surrounding biological milieu.^3^, ^4^, ^5^ Within this paradigm, the notion of biological inertness was considered advantageous: restorative materials were expected to remain stable and largely non-interactive, preserving their physicochemical properties in the presence of body fluids and helping maintain the structural integrity of the restored tooth.

However, clinical reality challenges this assumption. Restorations are frequently placed in substrates affected by caries, inflammation, enzymatic degradation, and altered mineral composition, where the interface is biologically active rather than inert.^7^, ^8^, ^9^, ^10^ In such scenarios, materials that merely “fill” defects may be insufficient to support long-term tissue stability, particularly in deep or compromised dentin, where repair mechanisms are continuously engaged.^10^, ^11^, ^12^ This context has also contributed to persistent misconceptions regarding what restorative “bioactivity” can realistically achieve in clinical practice, reinforcing the need for evidence-based interpretation rather than descriptive or marketing-driven use of the term.^4^, ^5^, ^6^

Importantly, this review does not interpret restorative bioactivity as synonymous with ion release or surface mineral precipitation alone. Throughout this manuscript, clinically relevant bioactivity is defined as the ability of restorative materials to produce measurable and functionally meaningful effects on the dentin-pulp complex, including preservation of dentin integrity, stabilization of the adhesive interface, modulation of inflammatory pathways, and support of tissue repair or regeneration under clinically relevant conditions^7^, ^13^, ^14^, ^15^ In this context, one of the major unresolved translational gaps in restorative dentistry remains the distinction between laboratory-demonstrated bioactivity and predictable long-term clinical functionality.

### Scope and evidence considerations

This narrative review synthesizes contemporary evidence on bioactive restorative materials. The discussion draws on peer-reviewed clinical and translational literature on ion-mediated effects, matrix stabilization strategies, vital pulp therapy materials, and emerging regenerative platforms. Priority is given to clinically relevant models and long-term functional outcomes, with the aim of critically distinguishing validated bioactivity from laboratory observations. The purpose is not to list ion-releasing materials, but rather to critically examine whether currently proposed bioactive mechanisms are supported by biologically and clinically meaningful evidence of influence on dentin-pulp preservation and restorative longevity.

### Bioactivity beyond the Buzzword: clarifying misconceptions in restorative dentistry

The intentional ability of a material to elicit specific, beneficial biological responses at the interface with dental tissues can be understood as bioactivity.^2^, ^4^, ^5^, ^6^ These responses extend beyond the material’s primary mechanical or restorative function. Historically, the concept of bioactivity emerged in the biomaterials field and was initially associated with the ability of materials to form apatite on their surface when in contact with biological fluids; however, contemporary interpretations in restorative dentistry have substantially broadened this scope.^4^, ^6^ Bioactivity today refers to a spectrum of chemical, mixed, or purely biological mechanisms capable of stimulating or directing cellular activity, promoting regenerative processes, modulating the dynamics of mineral content and organic matrix, or influencing microbial behavior.^2^, ^5^, ^15^

The term *bioactive material* has often been misused as a marketing label, applied to materials that merely release ions without evidence of clinically significant outcomes.^2^ In this sense, a central “myth” in restorative dentistry is the assumption that ion release alone necessarily translates into meaningful clinical functionality. True bioactivity in dentistry requires defined mechanisms, measurable biological effects, and translational validation, particularly because its clinical relevance lies in preserving and/or promoting repair/regeneration of dental tissues rather than simply demonstrating ion release in vitro.^2^, ^5^ Bioactive materials in dentistry, therefore, should be defined as restorative materials intentionally engineered to exert additional biological actions while still fulfilling their primary role of replacing lost tooth structure.

This review presents the scientific basis and clinical importance of developing and using bioactive materials in restorative dentistry, critically discussing the translational relevance of restorative bioactivity and the distinction between laboratory-demonstrated effects and clinically meaningful functionality at the dentin-pulp complex. Although enamel-focused bioactive strategies aimed at improving remineralization or preventing demineralization are recognized, this review emphasizes the dentin-pulp complex, where morphology, composition, and interaction exert dynamic functional effects that ultimately influence long-term clinical success. Table 1 summarizes the most common myths and the corresponding evidence-based realities discussed throughout this review.

**Table 1:** Common myths and evidence-based realities regarding bioactive restorative materials.

Myth (common misconception)	Evidence-based reality (according to current understanding)	Clinical implication
Myth 1: “Bioactivity” is defined simply by ion release.	Ion release alone does not necessarily translate into clinically meaningful functionality. True bioactivity requires defined mechanisms, measurable biological effects, and translational validation.^2^, ^5^, ^6^	Clinicians should not interpret “ion-releasing” as automatically beneficial without outcome-based evidence.
Myth 2: Surface apatite formation equals functional remineralization.	Many materials promote superficial or extrafibrillar mineral precipitation but fail to reliably achieve intrafibrillar remineralization within severely demineralized collagen matrices, particularly in caries-affected dentin.^2^, ^16^, ^17^, ^18^	Mineral deposition should not be assumed to restore dentin mechanical integrity or long-term stability.
Myth 3: All “bioactive” restorative materials are clinically equivalent.	Bioactivity encompasses a spectrum of physicochemical and biological mechanisms, and materials differ substantially in their secondary actions and level of clinical evidence support.^2^, ^5^, ^14^	Material selection should consider indication-specific evidence rather than generic labeling.
Myth 4: Demonstrating bioactivity in vitro is sufficient to claim clinical benefit.	Simplified experimental models do not reproduce the complexity of dentin-pulp interactions, pulpal pressure, biofilm challenge, aging, or inflammation. Clinically relevant interpretation requires substrate-relevant and translational validation.^15^, ^19^	Laboratory findings should be interpreted cautiously; clinically predictive models, together with longterm outcomes, remain essential.
Myth 5: Bioactivity is mainly about remineralization.	Restorative bioactivity extends beyond mineral recovery and may also involve collagen stabilization, inhibition of dentin proteases, modulation of cellular signaling pathways, and preservation of the adhesive interface.^7^, ^13^, ^14^, ^20^, ^21^, ^22^	Restoration longevity may depend as much on matrix preservation and interfacial stability as on mineral precipitation.
Myth 6: The dentin substrate is biologically inert.	Dentin acts as a biologically active reservoir of growth factors and signaling molecules released during demineralization, directly influencing pulp healing and reparative responses.^20^, ^23^, ^24^	Deep restorative procedures inherently interact with biological processes and therefore require biologically informed restorative strategies.
Myth 7: Vital pulp therapy materials achieve true regeneration.	Current pulp-capping systems, including calcium silicate-based bioceramics, primarily induce reparative outcomes rather than full regeneration, often relying on initial inflammatory or necrotic stimuli.^12^, ^25^, ^26^, ^27^	Clinicians should interpret VPT mainly as a strategy for tissue preservation and repair, whereas complete regeneration remains a future goal.
Myth 8: Scaffold-based regenerative systems are ready for clinical practice.	Although promising, scaffold-based regenerative strategies remain largely investigational due to translational challenges related to infection control, vascularization, delivery into complex anatomies, and regulatory feasibility.^19^, ^28^	Regenerative scaffold systems should currently be interpreted as future-oriented approaches rather than established clinical therapies.
Myth 9: The “bioactive” label guarantees long-term restoration longevity.	Durable clinical impact depends on well-defined mechanisms, workflow compatibility, substrate variability, and robust long-term validation rather than descriptive claims or short-term laboratory observations.^2^, ^14^, ^15^	Moving beyond labels requires focusing on evidence-supported functionality and predictable long-term clinical performance.

MMPs: matrix metalloproteinases; VPT: vital pulp therapy.

### The dentin-pulp complex as the primary target for restorative bioactivity

The dentin-pulp complex constitutes a functionally integrated biological system derived from a common mesenchymal origin and organized by mineralized and soft tissues linked by a specialized interface of cells.^20^, ^29^ Dentin forms the bulk of the tooth and displays a tubular architecture that allows diffusion and sensory transduction, while the pulp provides the vascular, neural, and cellular support for tissue homeostasis. Odontoblasts form a continuous cellular layer at this interface and extend cytoplasmic processes into the dentinal tubules, establishing a functional system in which structural integrity and biological activity are intrinsically linked.^20^

Dentin is not an inert substrate. Its extracellular matrix stores growth factors, non-collagenous proteins, and plasma-derived mediators that are released during demineralization events such as carious progression, cavity preparation, and adhesive conditioning.^6^, ^23^ (Table 2). These matrix-derived signals regulate odontoblast activity, recruit pulp progenitor cells, modulate angiogenesis, and support tissue repair, creating a dynamic feedback loop between matrix breakdown and biological signaling.^23^, ^24^ This property positions dentin as an active biological reservoir that directly influences pulp responses and healing, reinforcing conservative restorative approaches and the use of materials capable of preserving dentin-pulp homeostasis rather than merely replacing lost structure.

**Table 2:** Selected mineral constituents and extracellular matrix-associated components reported in dentin.

**Inorganic phase**			
Minerals	Major elements (wt%)	Calcium (Ca, 27.0%), phosphorus (P, 13.6%)	Hydroxyapatite crystals in the form of flattened plates (70 nm in length, 20-30 nm in width and 3-4 nm in thickness)
		Ca/P ratio (mol/mol) = 1.53	
	Trace elements (wt%)	Magnesium (Mg, 0.7%), sodium (Na, 0.9%), zinc (Zn, 0.01%), potassium (K, 0.03%), silicon (Si, 0.02%), strontium (Sr, 0.02%), chlorine (Cl, 0.06%), iron (Fe, 0.006%), copper (Cu, 0.001%), sulfur (S, 0.02%), fluoride (F, 0.02%)	
**Collagenous proteins**			
Collagens	Type I collagen	Most abundant protein in dentin ECM (*ca.* 90%)	
	Type III collagen	Only trace amounts	
	Type V collagen	Only trace amounts	
**Non-collagenous proteins (NCPs)**			
Proteoglycans (PGs)	Abbreviation/symbol	Full name	Main role(s)
	DCN	Decorin	Directing hydroxyapatite crystal growth
	BGN	Biglycan	Directing hydroxyapatite crystal growth
	LUM	Lumican	Limiting collagen fibril diameter and determining the regular spacing of fibrils
		Fibromodulin	Collagen fibrillogenesis control
	OSAD	Osteoadherin	Cell binding activity; high hydroxyapatite affinity
	VCAN	Versican	Maintenance of ECM hydration; formation of large complexes with hydroxyapatite and water
	-	Hyaluronan	Non-sulfated glycosaminoglycan involved in water binding, lubrication, extracellular matrix hydration, and macromolecular filtering.
Glycoproteins (GPs)	OCN	Osteocalcin (bone gamma-carboxyglutamate protein)	Tissue mineralization and calcium ion homeostasis; vitamin K-dependent
	SPARC or ON	Osteonectin (secreted protein acidic and cysteine rich)	Tissue mineralization, cell-matrix interactions, collagen binding and stimulation of MMP production
	SPP1 or OPN	Osteopontin (secreted phosphoprotein 1)	Cell recruitment to inflammatory sites, adhesion protein, involved in cell attachment and wound healing
	DMP1	Dentin matrix acidic phosphoprotein 1	Tissue mineralization, regulation of osteoblast-specific gene expression
	BSP	Bone sialoprotein (integrin binding sialoprotein)	Nucleation of apatite crystals
	DSP	Dentin sialoprotein	Regulation of initial dentin mineralization
	DPP	Dentin phosphoroprotein	Maturation of mineralized dentin
	DSPP	Dentin sialophosphoroprotein	Major NCP in dentin; critical for dentin mineralization; gives rise to DGP, DSP, and DPP
	MEPE	Matrix extracellular phosphoglycoprotein	Tissue mineralization
Serum proteins	ALB	Albumin	Not known
	IgG	Immunoglobulin G	Immune response to bacterial invasion
	TF	Transferrin	Iron-transport glycoprotein
	-	Fetuin-A	High affinity for hydroxyapatite. Inhibition of ectopic precipitation of calcium phosphate salts and control of MMP-2 and MMP-9 activities
Calcium binding proteins	CaM	Calmodulin	Calcium-binding protein; considered a major transducer of calcium signals
		Calbindin	Facilitates the vitamin D-dependent movement of calcium through the cytosolic compartment of the intestinal or renal cell
		Annexins	Aggregation and fusion of membrane vesicles, inhibition of blood coagulation, ion channel activity and inhibition of phospholipase A2
		Nucleobindin	Ca^2+^ sensor and buffer
Growth factors	IGF1	Insulin-like growth factor 1	Most potent activator of the AKT signaling pathway, a stimulator of cell growth and proliferation, and a potent inhibitor of apoptosis
	IGF2	Insulin-like growth factor 2	Growth-promoting hormone
	TGFB1	Transforming growth factor-beta 1	Performs many cellular functions, including cell growth, cell proliferation, cell differentiation, and apoptosis
	PDGF	Platelet-derived growth factor	Embryonic development, cell proliferation, cell migration, and angiogenesis. Required factor in cellular division for fibroblasts
	VEGF	Vascular endothelial growth factor A	Formation of blood vessels
	PGF	Placental growth factor	Member of the VEGF subfamily. Trophoblast growth and differentiation
	FGF2	Fibroblast growth factor 2	Proliferation and differentiation of several cell types
	EGF	Epidermal growth factor	Cell growth and differentiation
	ADM	Adrenomedullin	Angiogenesis, vasodilation, increases tolerance to oxidative stress and hypoxic injury
Enzymes (Proteases)	MMP-8	Matrix metalloproteinase 8 (collagenase 2)	Degradation of type I, II, and III collagens
	MMP-2	Matrix metalloproteinase 2 (gelatinase A)	Predominant MMP in sound dentin. Basement-membrane degradation. Degradation of gelatin, type I, II, IV, V, VII, and X collagens. Regulation of vascularization and inflammatory response
	MMP-9	Matrix metalloproteinase 9 (gelatinase B)	Cleavage of several matrix proteins such as decorin, biglycan, DSP, MEPE, BSP, and OPN
	MMP-20	Matrix metalloproteinase 20 (enamelysine)	
	MMP-3	Matrix metalloproteinase 3 (stromelysin-1)	
	MMP-13	Matrix metalloproteinase 13	
	MMP-14	Matrix metalloproteinase 14	
	CTSB	Cathepsin B	Cleavage of collagen molecules at multiple sites within the triple helix
	CTSK	Cathepsin K	Most potent mammalian collagenase
	ADAM/ADAMTS	Adamalysins	Collagen processing, cleavage of matrix proteoglycans, inhibition of angiogenesis, inhibition of blood coagulation homeostasis

Selected components and general biological roles compiled from references 7,20,30,31,32,33.

Carious injury disrupts this interface and triggers inflammatory and reparative cascades. When the insult remains distant from the pulp, surviving odontoblasts upregulate dentin deposition and form reactionary tertiary dentin, which reduces permeability and isolates the pulp.^24^, ^25^ With caries progression, odontoblast death occurs, and pulp stem and progenitor cells differentiate into odontoblast-like cells that produce reparative tertiary dentin as an attempt to create a mineralized barrier^24^, ^25^, ^34^ These pathways highlight the intrinsic reparative potential of the dentin-pulp complex, which remains highly dependent on the inflammatory microenvironment.^10^, ^25^, ^34^

Clinically, vital pulp therapy procedures, including direct pulp capping and pulpotomy, seek to preserve pulp vitality and stimulate tertiary dentin formation by applying capping and restorative materials directly to exposed or remaining vital pulp tissue.^26^ However, conventional calcium hydroxide- and calcium silicate-based materials primarily induce tissue repair rather than true regeneration, with superficial necrosis and less organized mineralized tissue being particularly associated with calcium hydroxide.^25^, ^27^ Moreover, deep carious lesions are frequently restored over caries-affected dentin, a substrate with reduced mineral content, altered collagen structure, increased enzymatic activity, and higher permeability, which compromises adhesive stability and accelerates interfacial degradation.^7^, ^8^, ^9^, ^11^

Together, these biological and chemical challenges redefine the dentin-pulp interface as a critical target for restorative materials that do more than seal defects: materials capable of actively modulating the microenvironment, supporting mineral stability, and promoting tissue preservation or regeneration. In this context, the clinical rationale for “bioactivity” must be interpreted beyond superficial mineral precipitation, emphasizing evidence-based functionality at the dentin-pulp complex.

### What restorative bioactivity can realistically deliver: mechanistic pathways with clinical relevance

Recognition of the dentin-pulp complex as a biologically responsive interface redefines the role of restorative materials. In deep and biologically compromised substrates, materials are no longer expected merely to seal cavities or replace lost structure, but to actively interact with dentin and pulp tissues by stabilizing and remineralizing the exposed collagen, controlling local inflammation, and guiding reparative and regenerative events.

**Figure 1:**
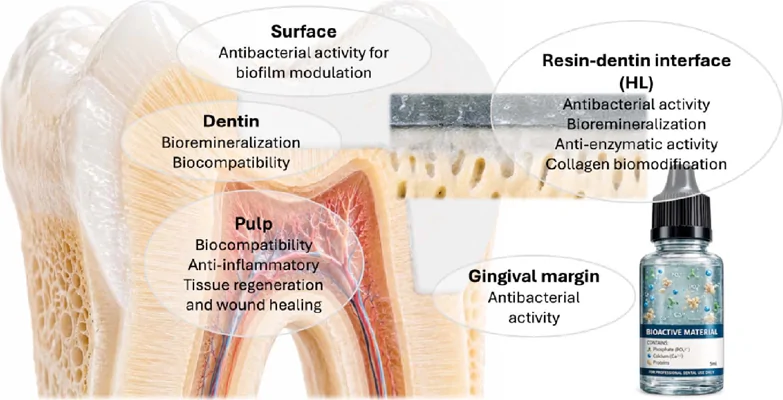
Desirable biological properties of restorative materials to extend the lifespan of dental restorations and preserve tooth structures. HL: hybrid layer.

Bioactivity in restorative dentistry can be broadly understood through interconnected physicochemical and biological pathways, rather than isolated laboratory phenomena.^6^ Importantly, these mechanisms should be interpreted in light of their potential to translate into clinical relevance, including dentin preservation, interfacial stability, pulp protection, and long-term restorative performance, although robust clinical evidence and long-term clinical trials remain limited for many currently proposed bioactive strategies. The desirable biological properties of restorative materials at different tissue interfaces are summarized in Figure 1.

### Ion-mediated effects: mineral recovery and environmental modulation

The presence of biologically relevant ions, including calcium (Ca^2+^), phosphate (PO_4_^3−^), and fluoride (F^−^), in the oral microenvironment is a main factor in inducing mineralization and promoting interactions between restorative materials and the dental substrate.^16^, ^17^, ^18^, ^35^, ^36^ This ion release occurs through physicochemical mechanisms governed by the material’s composition and by oral conditions, such as the presence of dentinal fluid and saliva. Once initiated, ion exchange increases local supersaturation and promotes mineral precipitation at the dentin interface, supporting remineralization and tissue repair.^16^

Bioactive materials containing calcium phosphate phases or other ion-releasing particles can promote the formation of amorphous calcium phosphate (ACP) precursor layers, which act as a transient ion reservoir and progressively transform into more stable apatite phases.^17^ From a clinical perspective, these reactions may contribute to partial mineral recovery and stabilization of demineralized dentin, although their ability to achieve true functional remineralization remains limited.^2^

Glass ionomer cements and related materials further contribute through sustained fluoride release. Fluoride primarily contributes to mineral recovery and control of caries progression by facilitating formation of fluoridated apatite and enhancing resistance to acid-mediated demineralization, while its direct antimicrobial effects are considered secondary under clinical conditions.^2^ Likewise, other therapeutic ions, such as silicate species from bioactive glasses and multifunctional ions (e.g., strontium and borate) derived from surface pre-reacted glass-ionomer (S-PRG) fillers, may support mineral deposition and broader tissue interactions at the restorative interface.^36^, ^37^

However, a central evidence-based limitation must be emphasized: although these materials promote surface mineral accumulation, they often fail to reliably induce intrafibrillar remineralization within severely demineralized collagen matrices, particularly in caries-affected dentin.^2^ Thus, ion release alone should not be equated with functional remineralization, which ideally involves recovery of intrafibrillar mineral content and structural stability of the collagen matrix;^2^, ^17^, ^18^ this represents one of the most common misconceptions surrounding restorative bioactivity.

In addition to mineral-related effects, pH modulation represents another relevant mechanism. Hydroxyl and calcium ions released from calcium hydroxide and calcium silicate-based systems create alkaline environments unfavorable for bacterial survival.^38^ Under these conditions, bacterial membranes and DNA may be damaged^38^ and endogenous mineralization pathways may be supported through alkaline phosphatase activation and calcium phosphate precipitation.^21^ Nevertheless, whether these effects persist under clinical conditions remains a key translational question.

### Dentin matrix preservation: collagen stability and enzymatic modulation

Maintaining the structural integrity of the dentin collagen matrix is essential for long-term restoration longevity, particularly at the resin-dentin interface. Demineralization exposes collagen fibrils to hydrolytic and enzymatic degradation, compromising the hybrid layer and accelerating interfacial failure.^7^, ^13^ Collagen biomodification strategies aim to prevent collagen breakdown by reinforcing the structural organization of the dentin matrix. Cross-linking agents strengthen collagen through intra- and intermolecular interactions, increasing matrix stiffness, reducing water sorption, and decreasing susceptibility to hydrolytic and enzymatic degradation.^7^, ^13^, ^14^ From a clinical perspective, these effects may contribute to improved hybrid-layer preservation and long-term adhesive stability.^7^, ^13^, ^14^

In parallel, inhibition of endogenous proteases, such as matrix metalloproteinases (MMPs) and cysteine cathepsins (CTs), reduces proteolytic activity within the dentin matrix, contributing to hybrid layer preservation.^7^, ^13^ Notably, ion-releasing platforms may also participate in enzymatic modulation. S-PRG eluates have been shown to significantly reduce dentin matrix-bound MMP activity and decrease collagen degradation over time, indicating that released ions may influence proteolysis beyond mineral precipitation.^39^

Together, these approaches illustrate that restorative bioactivity may extend beyond remineralization, targeting the preservation of the organic matrix as a prerequisite for durable interfacial stability.

### Cellular and signaling modulation toward dentinogenic responses

Beyond physicochemical interactions, dentin repair ultimately depends on cellular responses orchestrated by extracellular matrix proteins and odontogenic signaling pathways that regulate odontoblasts and pulp stem/progenitor cells.^20^, ^22^, ^32^ Odontoblasts synthesize a collagen-rich scaffold for mineral deposition, while dentin-specific non-collagenous proteins (NCPs), such as dentin matrix protein 1 (DMP-1) and dentin sialophosphoprotein (DSPP), regulate crystal nucleation and growth through their interactions with calcium and phosphate ions.^20^, ^32^

Importantly, restorative materials can influence these pathways by providing ionic cues that stimulate pulp cell proliferation, migration, and odontogenic differentiation, promoting the expression of key dentinogenic markers.^22^, ^40^, ^41^, ^42^, ^43^ Supporting this concept, transdentinal diffusion of ions released from S-PRG fillers has been shown to upregulate odontogenic markers (*Dspp, Dmpl, Collal*) and stimulate mineralization activity in odontoblast-like cells, reinforcing that ion release can exert biologically instructive effects beyond surface precipitation.^43^ Thus, clinically meaningful bioactivity should be interpreted not only as mineral deposition, but also as the modulation of cellular microenvironments toward biologically regulated mineralization responses.

### Evidence and experimental models: avoiding overinterpretation

The assessment of restorative bioactivity requires experimental models capable of capturing responses across increasing levels of complexity. In vitro systems provide valuable mechanistic insights into cytotoxicity, gene expression, enzymatic modulation, and mineralization outcomes.^8^, ^19^, ^43^

However, these simplified systems present important limitations, as they do not fully reproduce the complex biological interplay between dentin and pulp under clinical conditions. Therefore, transdentinal models provide a more clinically relevant approach by investigating the diffusion of bioactive components through dentin and their indirect effects on pulp cells, better approximating restorative scenarios in which materials act across the dentin barrier.^19^, ^43^ Ex vivo models using extracted human or animal teeth with preserved structural architecture allow direct analysis ofmaterial-dentin interactions, including ion diffusion through dentinal tubules and substrate-mediated biological effects.^19^ Ultimately, in vivo models remain essential to validate pulp repair, tertiary dentin formation, and inflammatory outcomes within functional physiological environments.^19^

Ongoing refinement of experimental protocols and the incorporation of advanced platforms, such as organ-on-a-chip and other physiologically relevant microfluidic models, will be essential to better align laboratory assessments of bioactivity with clinically meaningful performance and long-term outcomes.

## Bioactivity targeting dentin

Maintaining the structural integrity of dentinal collagen is essential for marginal sealing, remineralization, and long-term stability of the resin-dentin interface. Degradation of exposed collagen fibrils compromises the hybrid layer by increasing microleakage and ultimately reduces restoration longevity.^14^ Therefore, multiple strategies have been investigated to render the hybrid layer more resistant to hydrolytic and enzymatic degradation mediated by dentin proteases.^14^ These approaches include collagen biomodification through the use of cross-linking agents, inhibition of MMPs and CTs, antimicrobial approaches, hydrolysis-resistant materials, and biomimetic remineralization strategies.^14^ Together, these mechanisms may improve the durability of adhesive restorations.

### Remineralization strategies: surface precipitation versus functional recovery

**Figure 2:**
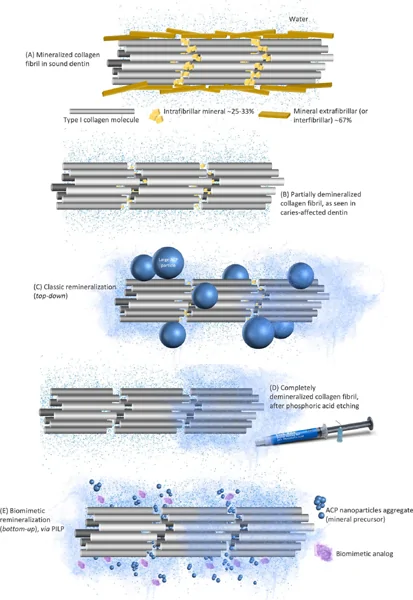
Schematic representation of the classic (top-down) and the biomimetic remineralization (bottom up) via polymer-induced liquid precursor (PILP) processes. (A) Mineralized collagen fibril found in sound dentin. Most minerals are located outside the fibrils (extrafibrillar minerals ~67%) while ~25 to 33% is packed inside the fibrils (intrafibrillar minerals), occupying the spaces between adjacent collagen molecules. (B) During the carious process, both extrafibrillar and intrafibrillar minerals are gradually dissolved, compromising the mechanical properties of dentine. However, the intrafibrillar remaining minerals serve as mineralizing sites, allowing epitaxial growth of the crystals in a thriving environment. (C) The classic remineralization process (top-down) involves the formation of large amorphous calcium phosphate (ACP) particles in the interfibrillar spaces, unable to reach the nanometric intrafibrillar spaces. (D) Phosphoric acid etching completely removes extrafibrillar and intrafibrillar collagen minerals, depleting the fibrils from remaining mineralizing sites. (E) The biomimetic remineralization aims to recover the intrafibrillar minerals by using non-collagenous dentin protein analogs to stabilize ACP nanoscale particles (mineral precursor ~2- 3 nm). These nanoparticles are small enough to fill the intrafibrillar spaces and serve as mineralizing sites, enabling controlled intrafibrillar mineral recovery beyond the superficial precipitation typically achieved by conventional ion-releasing materials.

The remineralization potential of dentin is closely dependent on the degree of residual mineral content. In partially demineralized substrates, such as caries-affected dentin, remaining intrafibrillar mineral may serve as nucleation sites for hydroxyapatite growth in the presence of calcium and phosphate ions.^35^ Conventional ion-releasing materials, including glass ionomer cements and calcium silicate-based systems, can promote predominantly extrafibrillar mineral precipitation, often described as a “top-down” remineralization pattern, which represents the most common clinical manifestation of ion-driven bioactivity at the dentin interface (Figure 2).^2^, ^18^

In severely demineralized collagen matrices, biomimetic (“bottom-up”) remineralization approaches rely on protein-inspired analogs to guide mineral precursor infiltration and crystal growth within collagen fibrils.^2^, ^18^ Biomimetic remineralization systems commonly employ analogs of non-collagenous proteins, such as polyacrylic acid or polyaspartic acid, which stabilize amorphous calcium phosphate nanophases, enabling controlled intrafibrillar mineral recovery beyond the superficial precipitation typically achieved by conventional ion-releasing materials.^2^, ^18^ This process aims to replace water-filled spaces within collagen with mineral, reducing hydrolytic breakdown, limiting proteolytic susceptibility, and potentially improving long-term bond stability.^2^, ^18^ From a translational perspective, incorporation of these biomimetic components into restorative systems, such as ionomer-based materials or adhesive primers, represents one of the most promising routes toward clinically applicable remineralizing therapies.^2^, ^18^

Bioactive glasses have been extensively investigated as dentin-targeted remineralizing additives, primarily because their dissolution pro ducts can support apatite-forming reactions and local mineral recovery in mineral-depleted dentin.^36^ Their compositions can be tailored with specific dopants to optimize physicochemical and biological performance. Similarly, surface pre-reacted glass-ionomer (S-PRG) fillers represent a multifunctional platform, providing multiple ions (e.g., fluoride, strontium, borate, and silicate) that may contribute not only to mineral recovery but also to acid neutralization, antimicrobial effects, and enzymatic modulation.^37^, ^39^, ^43^ Calcium phosphate reservoirs such as CPP-ACP have also been incorporated into restorative systems to sustain ion availability and facilitate mineral precipitation in partially demineralized dentin substrates.^17^

Rather than representing distinct categories, these ion-based systems share a common translational goal: supporting mineral recovery while simultaneously influencing the biological stability of the dentin interface. Nevertheless, mineral deposition should not be automatically interpreted as functional remineralization. An important translational question remains whether these approaches consistently achieve true intrafibrillar mineral restoration within the collagen matrix, or whether their effects are largely limited to superficial precipitation on the dentin surface. Clarifying this distinction is essential for interpreting the long-term clinical relevance of “remineralizing” restorative systems.

### Collagen biomodification and hybrid-layer stabilization

Given the enzymatic vulnerability of the hybrid layer, clinically oriented strategies have focused on delivering collagen-stabilizing and protease-inhibiting effects through adhesive pretreatments, primers, or restorative formulations.^13^, ^14^ Cross-linking agents may be synthetic (e.g., glutaraldehyde, carbodiimide) or naturally derived polyphenols, such as proanthocyanidins and flavonoids, which have gained interest due to improved biocompatibility and multifunctional properties.^13^, ^14^ These compounds can be applied as pretreatments or incorporated into adhesive systems, with concentration-dependent effects that require a careful balance between efficacy and cytocompatibility.^13^, ^14^

From a clinical perspective, the main challenge is not the proof-of-concept of collagen reinforcement but the development of protocols that are compatible with restorative workflows, provide durable effects after aging, and remain effective across substrate variability. Accordingly, simplified delivery strategies, such as incorporation into universal adhesives or multifunctional restorative formulations, are increasingly explored as more realistic pathways for clinical adoption.

In this context, recent evidence has demonstrated that pretreatment of dentin with grape seed extract, delivered in simplified solvent systems, can enhance collagen stability and significantly reduce nanoleakage even under dry-bonding conditions, mitigating technique sensitivity and improving interfacial integrity.^44^, ^45^

Although collagen biomodification has demonstrated potential to preserve bond strength over aging in sound dentin, outcomes remain substrate-dependent. Caries-affected, eroded, and radicular dentin continue to represent challenging environments where long-term stability may be limited. Thus, the clinical effectiveness of these approaches likely depends not only on the cross-linking chemistry itselfbut also on the pathological microenvironment in which the adhesive interface forms, reinforcing the need for substrate-specific translational validation.^14^

## Bioactivity targeting pulp

Bioactive strategies in restorative dentistry extend beyond dentin remineralization and adhesive interface stabilization, reaching the preservation and modulation of the pulp tissue itself. Because dentin and pulp form a biologically interdependent complex, deep restorative interventions inevitably influence the pulp microenvironment through the dentinal tubular network, even in the absence of direct exposure. Therefore, maintaining pulp vitality is central to sustaining continuous dentin deposition, immune-inflammatory balance, vascular supply, and long-term tooth survival.^28^

In this review, reparative responses refer primarily to tertiary dentin deposition and tissue preservation following injury, whereas regeneration implies the reestablishment of a functional dentin-pulp complex with organized cellular, vascular, neural, and extracellular matrix architecture. Although these concepts are frequently used interchangeably in the literature, they represent biologically distinct outcomes with substantially different translational implications.^25^

Within this context, the classical paradigm of restorative materials as inert substitutes has progressively shifted. Contemporary biomaterials are increasingly expected not only to seal defects and replace lost structure, but also to exert biomodulatory effects that control inflammation and support reparative dentinogenesis. Among the various proposed applications of restorative bioactivity, vital pulp therapy represents the most clinically established and evidence-supported scenario.

### Pulp-capping materials and reparative dentinogenesis

Vital pulp therapy (VPT) constitutes the most clinically established application of pulp-directed bioactivity. Caries-induced injury triggers inflammatory cascades mediated by odontoblasts, fibroblasts, immune cells, and pulp stem/progenitor populations. These cells express pattern-recognition receptors, including Toll-like receptors, which activate downstream signaling pathways (e.g., NF-κB and MAPKs) and regulate cytokine release, antimicrobial responses, and innate immune defenses.^10^

Pulp inflammation is increasingly understood as a dynamic event rather than an irreversible endpoint.^12^ Controlled inflammatory signaling may stimulate repair and regeneration, whereas persistent or excessive inflammation compromises healing and leads to necrosis.^10^ This evolving biological understanding has supported more conservative clinical approaches, including selective caries removal and VPT procedures, even in cases previously considered unsuitable for pulp preservation.^12^ From a biomaterials perspective, the objective of pulp-capping and VPT materials is not only to provide sealing but also to create a microenvironment that promotes odontoblast-like differentiation, mineralized barrier formation, and resolution of inflammation.^28^ Calcium hydroxide has historically been the reference material due to its high alkalinity, which induces superficial coagulation necrosis followed by recruitment of mesenchymal progenitor cells and dentin bridge formation.^25^, ^27^ However, calcium hydroxide presents limitations such as solubility, poor long-term sealing, and the biological cost of considerable tissue injury.^25^, ^27^

Consequently, hydraulic calcium silicate-based bioceramics, including mineral trioxide aggregate (MTA) and related formulations, are currently regarded as first-line materials for direct pulp capping.^12^ However, in selected clinical situations involving preserved dentin thickness and controlled pulpal conditions, glass ionomer cements and resin-based restorative materials may also be placed without requiring liner application when adequate dentin thickness and pulpal conditions are preserved.^25^

Hydraulic calcium silicate-based systems provide mechanisms similar to those of calcium hydroxide, but with sustained calcium ion release, improved dimensional stability, and superior sealing ability, supporting reparative dentinogenesis and more favorable pulp outcomes.^12^ As their dissolution products can stimulate mineral deposition and modulate pulp cell behavior, VPT materials function as biologically interactive systems rather than passive barriers. Nevertheless, even contemporary bioceramic cements remain largely reparative (tertiary dentin formation and tissue preservation) rather than truly regenerative (reestablishment of a functional dentin-pulp complex), often involving an initial inflammatory response, with superficial necrosis being particularly associated with calcium hydroxide.^25^, ^26^ In addition, materials such as MTA and related calcium silicate-based cements still carry significant clinical limitations, including potential discoloration, technique sensitivity, and variability in outcomes depending on case selection and pulpal inflammatory status.^12^, ^26^ This highlights the ongoing need for next-generation pulp-capping biomaterials capable of more precisely modulating inflammation and minimizing early tissue damage.

### Emerging regenerative perspectives

Beyond VPT, scaffold-based regenerative strategies represent an emerging frontier aimed at restoring functional pulp tissue. Tissue engineering approaches are classically based on the integration of scaffolds, stem/progenitor cells, and bioactive signaling molecules, designed to recreate a microenvironment that supports tissue regeneration.^22^, ^28^

Among the proposed platforms, electrospun nanofibrous scaffolds have gained attention due to their extracellular matrix-like architecture, supporting odontogenic differentiation and mineralized tissue formation in dentin-pulp regenerative models.^22^, ^40^, ^41^, ^42^, ^46^, ^47^, ^48^, ^49^, ^50^ A wide range of biomaterials has been explored, including natural polymers with intrinsic bioactivity (e.g., collagen-, gelatin-, or chitosan-based matrices) and synthetic systems (e.g., polyester-based platforms) that offer greater tunability in degradation rate, mechanical behavior, and drug delivery performance.^22,28^ These scaffolds may incorporate growth factors, chemotactic signals, immunomodulatory molecules, or ion-releasing components to enhance regenerative outcomes.^22^, ^28^, ^46^, ^47^, ^48^, ^49^, ^50^

However, despite promising experimental evidence, scaffold-based regenerative systems remain largely investigational. Key translational challenges include infection control, vascularization of regenerated tissue, delivery into irregular pulp spaces, and regulatory feasibility. Thus, scaffold-based bioactivity should currently be interpreted as a future-oriented strategy rather than an established component of routine clinical restorative practice.

## Clinical validation as the key challenge for bioactive restoratives

Despite major advances in the development of ion-releasing, collagen-stabilizing, and regenerative-inspired restorative systems, the translation ofbioactivity into consistent clinical benefit remains one of the central challenges in contemporary restorative dentistry. The dentin-pulp complex represents a dynamic target: dentin is simultaneously a structural substrate, a diffusion barrier, and a biological reservoir, while the pulp is a vascularized and immunologically active tissue whose response is highly dependent on inflammation, bacterial control, and substrate condition.

Therefore, restorative bioactivity cannot be interpreted only through isolated mineral precipitation or short-term cellular outcomes. Instead, it must be validated within clinically realistic environments and supported by long-term clinical evidence of meaningful improvements in restorative performance and dentin–pulp preservation.

A critical limitation in the field is the overinterpretation of in vitro ion release or apatite formation as evidence of long-term functional benefit. In many systems, mineral deposition may remain superficial or extrafibrillar, without restoring the mechanical integrity of the collagen matrix or preventing long-term enzymatic degradation. Similarly, biochemical modulation, such as reductions in MMP activity or upregulation of odontogenic markers, does not necessarily translate into durable interfacial stability or predictable pulp outcomes under clinical conditions. Thus, one of the most persistent myths surrounding restorative bioactivity is the assumption that laboratory-demonstrated effects automatically equate to clinically meaningful functionality. The distinction between proof-of-concept bioactivity and validated clinical performance remains fundamental.

From a translational perspective, the success of bioactive restorative strategies will depend on substrate-relevant testing models, workflow-compatible delivery approaches, and long-term clinical validation demonstrating improved interfacial stability, dentin-pulp preservation, and compatibility with routine restorative procedures.^14^, ^15^, ^25^, ^28^ Clinically meaningful translation will ultimately require consistent performance under conditions involving pulpal pressure, biofilm challenge, aging, and substrate variability.^8^, ^14^, ^15^, ^28^

In addition, scaffold-based regenerative systems illustrate both the promise and the current limitations of pulp-directed bioactivity. Despite encouraging experimental evidence, clinical translation remains constrained by challenges including infection control, vascularization, delivery into complex anatomies, and regulatory feasibility. Accordingly, regenerative scaffold strategies remain largely investigational, despite their significant therapeutic potential.

Ultimately, moving beyond the *bioactive* label will require a shift from descriptive demonstrations toward restorative systems that reliably preserve dentin integrity, stabilize the interface, control inflammation, and support long-term dentin-pulp homeostasis.

## Conclusion

Bioactivity in restorative dentistry has expanded from a concept of apatite formation to a broader framework in which materials are expected to actively interact with the dentin–pulp complex. As discussed throughout this review, ion release, collagen stabilization, enzymatic inhibition, and emerging regenerative strategies may modulate mineral dynamics, cellular responses, and inflammatory balance at the tissue interface. However, a critical challenge remains in distinguishing biologically relevant effects from superficial precipitation or short-term in vitro observations. Therefore, clinically meaningful translation will depend on well-defined mechanisms, substrate-relevant models, and long-term validation. Bioactivity must be understood as more than a trend or marketing label: its rationale in clinical practice lies in developing restorative systems that go beyond replacement to reliably support interface stability and dentin–pulp homeostasis over time.

## Data Availability

The authors declare that all data generated or analyzed during this study are included in this published article.
